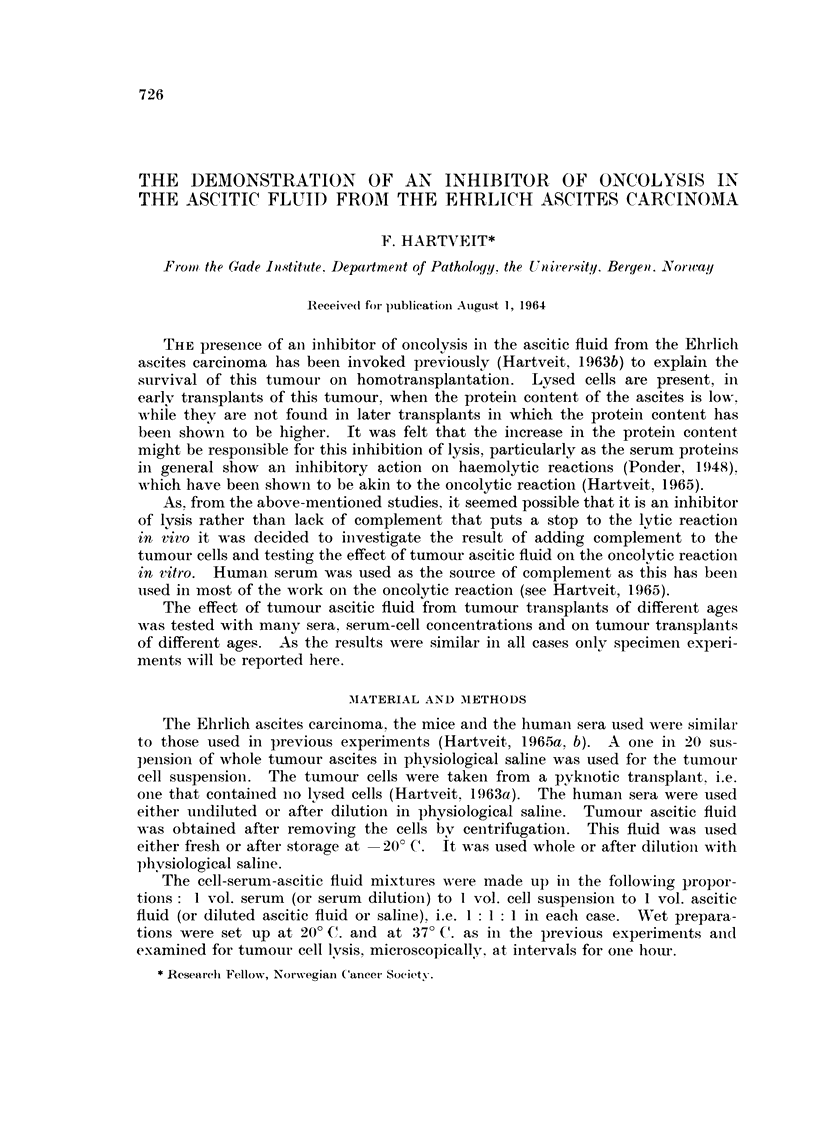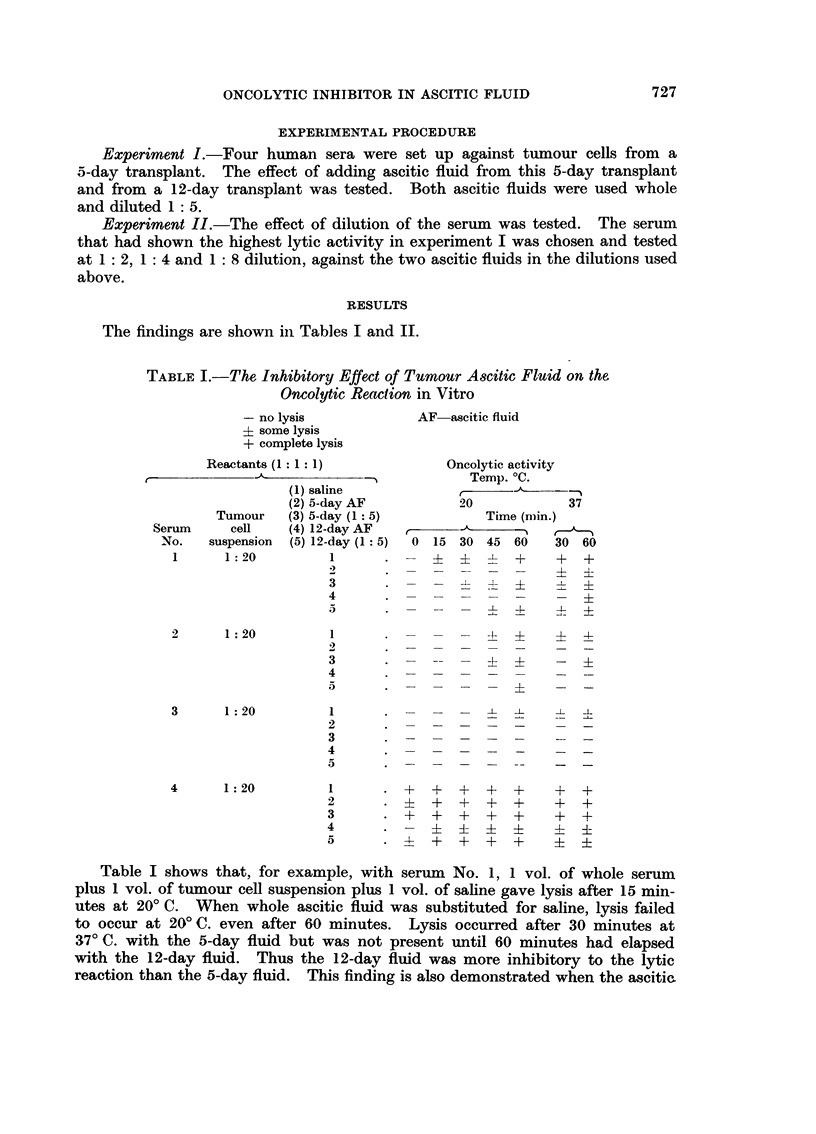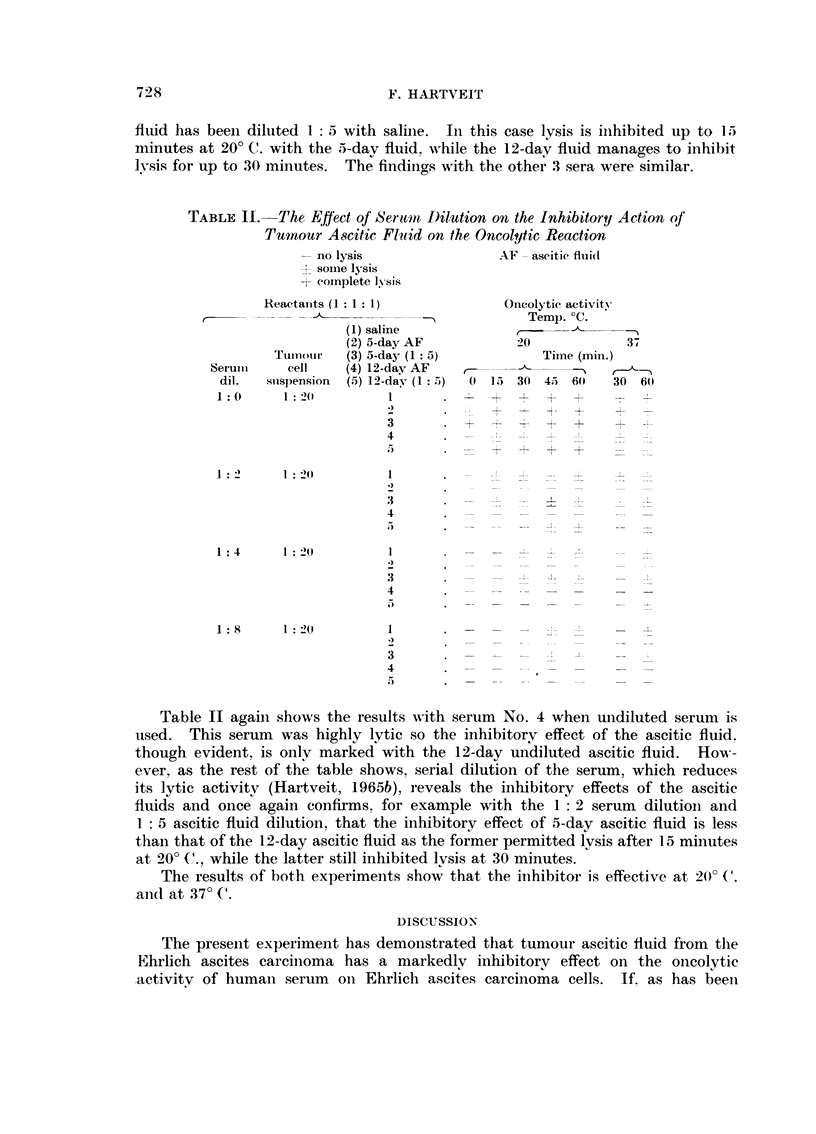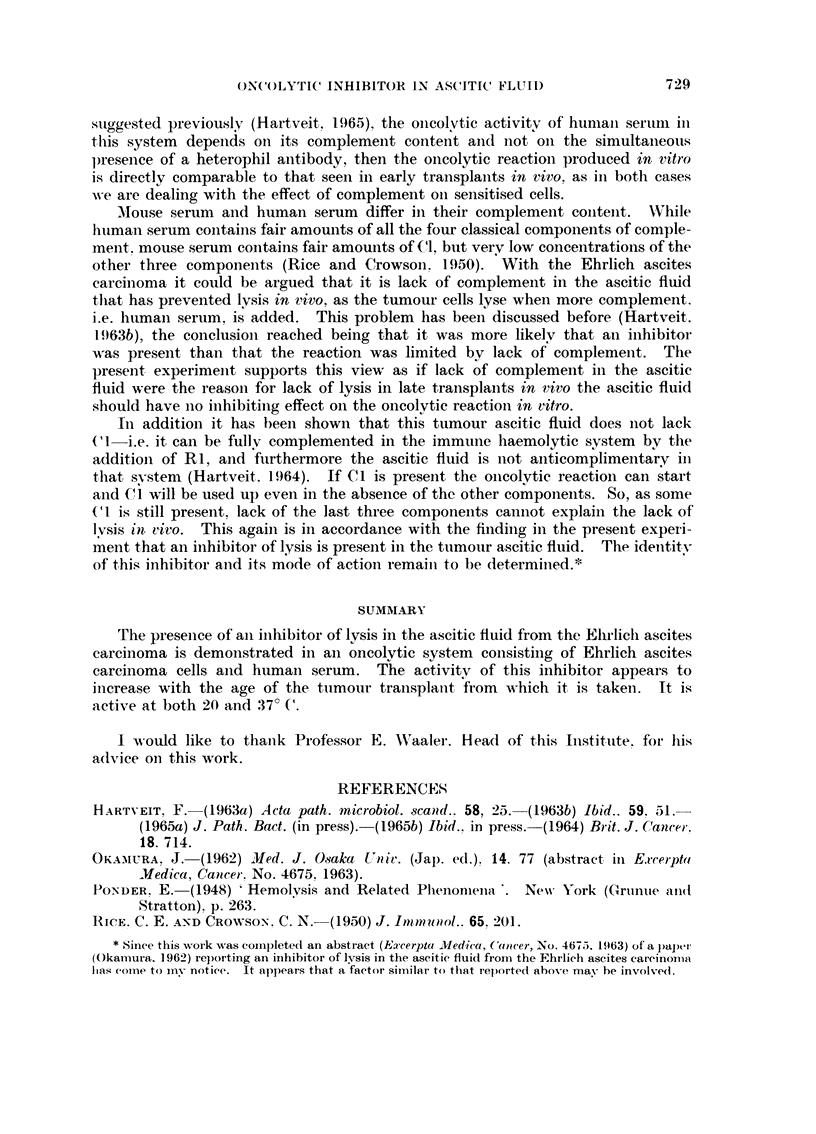# The Demonstration of an Inhibitor of Oncolysis in the Ascitic Fluid from the Ehrlich Ascites Carcinoma

**DOI:** 10.1038/bjc.1964.84

**Published:** 1964-12

**Authors:** F. Hartveit


### THE DEMONSTRATION OF AN INHIBITOR OF ONCOLYSIS IN THE ASCITIC FLUID FROM THE EHRLICH ASCITES CARCINOMA

F. HARTVEIT*

*From the Gade Institute, Department of Pathology, the University, Bergen, Norway*

Received for publication August 1, 1964

THE presence of an inhibitor of oncolysis in the ascitic fluid from the Ehrlich ascites carcinoma has been invoked previously (Hartveit, 1963*b*) to explain the survival of this tumour on homotransplantation. Lysed cells are present, in early transplants of this tumour, when the protein content of the ascites is low, while they are not found in later transplants in which the protein content has been shown to be higher. It was felt that the increase in the protein content might be responsible for this inhibition of lysis, particularly as the serum proteins in general show an inhibitory action on haemolytic reactions (Ponder, 1948), which have been shown to be akin to the oncolytic reaction (Hartveit, 1965).

As, from the above-mentioned studies, it seemed possible that it is an inhibitor of lysis rather than lack of complement that puts a stop to the lytic reaction *in vivo* it was decided to investigate the result of adding complement to the tumour cells and testing the effect of tumour ascitic fluid on the oncolytic reaction *in vitro*. Human serum was used as the source of complement as this has been used in most of the work on the oncolytic reaction (see Hartveit, 1965).

The effect of tumour ascitic fluid from tumour transplants of different ages was tested with many sera, serum-cell concentrations and on tumour transplants of different ages. As the results were similar in all cases only specimen experiments will be reported here.

### MATERIAL AND METHODS

The Ehrlich ascites carcinoma, the mice and the human sera used were similar to those used in previous experiments (Hartveit, 1965*a*, *b*). A one in 20 suspension of whole tumour ascites in physiological saline was used for the tumour cell suspension. The tumour cells were taken from a pyknotic transplant, i.e. one that contained no lysed cells (Hartveit, 1963*a*). The human sera were used either undiluted or after dilution in physiological saline. Tumour ascitic fluid was obtained after removing the cells by centrifugation. This fluid was used either fresh or after storage at −20° C. It was used whole or after dilution with physiological saline.

The cell-serum-ascitic fluid mixtures were made up in the following proportions : 1 vol. serum (or serum dilution) to 1 vol. cell suspension to 1 vol. ascitic fluid (or diluted ascitic fluid or saline), i.e. 1 : 1 : 1 in each case. Wet preparations were set up at 20° C. and at 37° C. as in the previous experiments and examined for tumour cell lysis, microscopically, at intervals for one hour.

* Research Fellow, Norwegian Cancer Society.

### EXPERIMENTAL PROCEDURE

*Experiment I.*—Four human sera were set up against tumour cells from a 5-day transplant. The effect of adding ascitic fluid from this 5-day transplant and from a 12-day transplant was tested. Both ascitic fluids were used whole and diluted 1 : 5.

*Experiment II.*—The effect of dilution of the serum was tested. The serum that had shown the highest lytic activity in experiment I was chosen and tested at 1 : 2, 1 : 4 and 1 : 8 dilution, against the two ascitic fluids in the dilutions used above.

### RESULTS

The findings are shown in Tables I and II.

TABLE I.—*The Inhibitory Effect of Tumour Ascitic Fluid on the Oncolytic Reaction* in Vitro

− no lysis
± some lysis
+ complete lysis

AF—ascitic fluid

| Reactants (1 : 1 : 1) | | | Oncolytic activity Temp. °C. | | | | | | |
|---|---|---|---|---|---|---|---|---|---|
| | | | 20 | | | | | 37 | |
| | | | Time (min.) | | | | | | |
| Serum No. | Tumour cell suspension | (1) saline (2) 5-day AF (3) 5-day (1 : 5) (4) 12-day AF (5) 12-day (1 : 5) | 0 | 15 | 30 | 45 | 60 | 30 | 60 |
| 1 | 1 : 20 | 1 | − | ± | ± | ± | + | + | + |
| | | 2 | − | − | − | − | − | ± | ± |
| | | 3 | − | − | ± | ± | ± | ± | ± |
| | | 4 | − | − | − | − | − | − | ± |
| | | 5 | − | − | − | ± | ± | ± | ± |
| 2 | 1 : 20 | 1 | − | − | − | ± | ± | ± | ± |
| | | 2 | − | − | − | − | − | − | − |
| | | 3 | − | − | − | ± | ± | − | ± |
| | | 4 | − | − | − | − | − | − | − |
| | | 5 | − | − | − | − | ± | − | − |
| 3 | 1 : 20 | 1 | − | − | − | ± | ± | ± | ± |
| | | 2 | − | − | − | − | − | − | − |
| | | 3 | − | − | − | − | − | − | − |
| | | 4 | − | − | − | − | − | − | − |
| | | 5 | − | − | − | − | − | − | − |
| 4 | 1 : 20 | 1 | + | + | + | + | + | + | + |
| | | 2 | ± | + | + | + | + | + | + |
| | | 3 | + | + | + | + | + | + | + |
| | | 4 | − | ± | ± | ± | ± | ± | ± |
| | | 5 | ± | + | + | + | + | ± | ± |

Table I shows that, for example, with serum No. 1, 1 vol. of whole serum plus 1 vol. of tumour cell suspension plus 1 vol. of saline gave lysis after 15 minutes at 20° C. When whole ascitic fluid was substituted for saline, lysis failed to occur at 20° C. even after 60 minutes. Lysis occurred after 30 minutes at 37° C. with the 5-day fluid but was not present until 60 minutes had elapsed with the 12-day fluid. Thus the 12-day fluid was more inhibitory to the lytic reaction than the 5-day fluid. This finding is also demonstrated when the ascitic fluid has been diluted 1 : 5 with saline. In this case lysis is inhibited up to 15 minutes at 20° C. with the 5-day fluid, while the 12-day fluid manages to inhibit lysis for up to 30 minutes. The findings with the other 3 sera were similar.

TABLE II.—*The Effect of Serum Dilution on the Inhibitory Action of Tumour Ascitic Fluid on the Oncolytic Reaction*

— no lysis
± some lysis
+ complete lysis
AF – ascitic fluid

| Reactants (1 : 1 : 1) | | | Oncolytic activity Temp. °C. | | | | | | |
|---|---|---|---|---|---|---|---|---|---|
| | | | 20 | | | | | 37 | |
| | | | Time (min.) | | | | | | |
| Serum dil. | Tumour cell suspension | (1) saline (2) 5-day AF (3) 5-day (1 : 5) (4) 12-day AF (5) 12-day (1 : 5) | 0 | 15 | 30 | 45 | 60 | 30 | 60 |
| 1 : 0 | 1 : 20 | 1 . | + | + | + | + | + | + | + |
| | | 2 . | ± | + | + | + | + | + | + |
| | | 3 . | + | + | + | + | + | + | + |
| | | 4 . | — | ± | ± | ± | ± | ± | ± |
| | | 5 . | ± | + | + | + | + | ± | ± |
| 1 : 2 | 1 : 20 | 1 . | — | ± | ± | ± | ± | ± | ± |
| | | 2 . | — | — | — | — | — | — | — |
| | | 3 . | — | ± | ± | ± | ± | ± | ± |
| | | 4 . | — | — | — | — | — | — | — |
| | | 5 . | — | — | — | ± | ± | — | ± |
| 1 : 4 | 1 : 20 | 1 . | — | — | ± | ± | ± | — | ± |
| | | 2 . | — | — | — | — | — | — | — |
| | | 3 . | — | — | ± | ± | ± | — | ± |
| | | 4 . | — | — | — | — | — | — | — |
| | | 5 . | — | — | — | — | — | — | ± |
| 1 : 8 | 1 : 20 | 1 . | — | — | — | ± | ± | — | ± |
| | | 2 . | — | — | — | — | — | — | — |
| | | 3 . | — | — | — | ± | ± | — | ± |
| | | 4 . | — | — | — | — | — | — | — |
| | | 5 . | — | — | — | — | — | — | — |

Table II again shows the results with serum No. 4 when undiluted serum is used. This serum was highly lytic so the inhibitory effect of the ascitic fluid, though evident, is only marked with the 12-day undiluted ascitic fluid. However, as the rest of the table shows, serial dilution of the serum, which reduces its lytic activity (Hartveit, 1965*b*), reveals the inhibitory effects of the ascitic fluids and once again confirms, for example with the 1 : 2 serum dilution and 1 : 5 ascitic fluid dilution, that the inhibitory effect of 5-day ascitic fluid is less than that of the 12-day ascitic fluid as the former permitted lysis after 15 minutes at 20° C., while the latter still inhibited lysis at 30 minutes.

The results of both experiments show that the inhibitor is effective at 20° C. and at 37° C.

### DISCUSSION

The present experiment has demonstrated that tumour ascitic fluid from the Ehrlich ascites carcinoma has a markedly inhibitory effect on the oncolytic activity of human serum on Ehrlich ascites carcinoma cells. If, as has been suggested previously (Hartveit, 1965), the oncolytic activity of human serum in this system depends on its complement content and not on the simultaneous presence of a heterophil antibody, then the oncolytic reaction produced *in vitro* is directly comparable to that seen in early transplants *in vivo*, as in both cases we are dealing with the effect of complement on sensitised cells.

Mouse serum and human serum differ in their complement content. While human serum contains fair amounts of all the four classical components of complement, mouse serum contains fair amounts of C1, but very low concentrations of the other three components (Rice and Crowson, 1950). With the Ehrlich ascites carcinoma it could be argued that it is lack of complement in the ascitic fluid that has prevented lysis *in vivo*, as the tumour cells lyse when more complement, i.e. human serum, is added. This problem has been discussed before (Hartveit, 1963*b*), the conclusion reached being that it was more likely that an inhibitor was present than that the reaction was limited by lack of complement. The present experiment supports this view as if lack of complement in the ascitic fluid were the reason for lack of lysis in late transplants *in vivo* the ascitic fluid should have no inhibiting effect on the oncolytic reaction *in vitro.*

In addition it has been shown that this tumour ascitic fluid does not lack C1—i.e. it can be fully complemented in the immune haemolytic system by the addition of R1, and furthermore the ascitic fluid is not anticomplimentary in that system (Hartveit, 1964). If C1 is present the oncolytic reaction can start and C1 will be used up even in the absence of the other components. So, as some C1 is still present, lack of the last three components cannot explain the lack of lysis *in vivo.* This again is in accordance with the finding in the present experiment that an inhibitor of lysis is present in the tumour ascitic fluid. The identity of this inhibitor and its mode of action remain to be determined.*

SUMMARY

The presence of an inhibitor of lysis in the ascitic fluid from the Ehrlich ascites carcinoma is demonstrated in an oncolytic system consisting of Ehrlich ascites carcinoma cells and human serum. The activity of this inhibitor appears to increase with the age of the tumour transplant from which it is taken. It is active at both 20 and 37° C.

I would like to thank Professor E. Waaler, Head of this Institute, for his advice on this work.

### REFERENCES

HARTVEIT, F.—(1963*a*) *Acta path. microbiol. scand.*, **58**, 25.—(1963*b*) *Ibid.*, **59**, 51.—(1965*a*) *J. Path. Bact.* (in press).—(1965*b*) *Ibid.*, in press.—(1964) *Brit. J. Cancer*, **18**, 714.

OKAMURA, J.—(1962) *Med. J. Osaka Univ.* (Jap. ed.), **14**, 77 (abstract in *Excerpta Medica, Cancer*, No. 4675, 1963).

PONDER, E.—(1948) 'Hemolysis and Related Phenomena'. New York (Grunue and Stratton), p. 263.

RICE, C. E. AND CROWSON, C. N.—(1950) *J. Immunol.*, **65**, 201.

* Since this work was completed an abstract (*Excerpta Medica, Cancer*, No. 4675, 1963) of a paper (Okamura, 1962) reporting an inhibitor of lysis in the ascitic fluid from the Ehrlich ascites carcinoma has come to my notice. It appears that a factor similar to that reported above may be involved.